# Core outcome domains for *Mycobacterium avium* complex pulmonary disease: a MACCOR study

**DOI:** 10.1183/23120541.00636-2025

**Published:** 2025-12-22

**Authors:** Cara D. Varley, Sarah A.R. Siegel, Naomi A. DeBacker, Clifton O. Bingham, Alexandra L. Quittner, David M. Lewinsohn, Luke Strnad, Kevin L. Winthrop

**Affiliations:** 1 Oregon Health and Science University, School of Medicine, Portland, OR, USA; 2 Oregon Health and Science University–Portland State University School of Public Health, Portland, OR, USA; 3 Johns Hopkins University, School of Medicine, Baltimore, MD, USA; 4 Joe DiMaggio Cystic Fibrosis, Pulmonary and Sleep Center, Hollywood, FL, USA; 5 Veterans Affairs Portland Health Care System, Portland, OR, USA

## Abstract

**Background:**

*Mycobacterium avium complex* pulmonary disease (MAC-PD) is a chronic inflammatory disease with systemic manifestations affecting multiple aspects of patients’ lives. Current microbiological outcome measures are often difficult to obtain and insufficient to reflect the impact of disease and treatment. We developed an international consensus-based set of core outcome domains (COD) for MAC-PD clinical trials using a modified Delphi consensus methodology.

**Methods:**

We invited relevant stakeholders in MAC-PD including people living with MAC-PD, their family members and friends, clinicians, researchers and research funding organisations to participate in an electronically administered Delphi consensus process. Participants rated preliminary domain importance, without regard to availability, feasibility or validity of potential measurement instruments. We used descriptive analyses to evaluate participant demographics and domain ratings, with predetermined criteria for COD inclusion.

**Results:**

A total of 306 participants representing 17 countries participated in round 1, 197 (64.4%) in round 2 and 173 (56.5%) in round 3. Over half of participants were people living with MAC-PD (57.2%), with 31.1% reporting their primary role as clinician, 9.2% as researcher. Symptoms, microbiology, treatment side-effects, chest imaging, physical function, treatment burden, and vitality/energy domains met criteria for COD inclusion in round 1. Disease recurrence and biomarkers met COD inclusion criteria in round 2 and round 3, respectively.

**Conclusion:**

Participants evaluated 11 outcome domains and suggested two additional domains for consideration, reaching COD consensus inclusion criteria for nine domains. Identification and dissemination of these CODs will help guide research priorities for measurement instruments and facilitate composite measure of disease activity development for MAC-PD.

## Shareable abstract

**Participants reached consensus for nine core outcome domains for MAC-PD: symptoms, microbiology, treatment side-effects, chest imaging, physical function, treatment burden, vitality/energy, disease recurrence and biomarkers** https://bit.ly/43LHX6J

## Introduction

Pulmonary nontuberculous mycobacteria (NTM) disease, most commonly *Mycobacterium avium* complex (MAC), is an orphan disease with increasing prevalence and incidence [1, 2]. MAC pulmonary disease (MAC-PD) is a chronic inflammatory airway disease, with frequent relapses and recurrence, associated with chronic cough, fatigue, night sweats, weight loss and depression [2–4]. In contrast to many acute infections, but similar to other chronic inflammatory diseases, the impact of MAC-PD varies between individuals in terms of presentation, course and response to therapy.

The American Thoracic Society and Infectious Diseases Society of America recommend using the triad of sputum culture, radiographic characteristics, and symptoms to drive treatment decisions, but validated measurement instruments of these categories for MAC-PD are not available, and as a result, management approaches vary widely [3–6]. When treatment is initiated, patients with MAC-PD typically take between three and five antibiotics for 18–24 months [3, 4]. Culture conversion is currently the gold standard for treatment success and is our only direct measure of a potential treatment's mycobacterial effect [3, 4]. However, culture conversion is often difficult to confirm if a patient cannot produce a sputum sample adequate for mycobacterial culture, even with procedures such as sputum induction, after being treated. Conversely, the implications of persistently positive cultures in a patient with symptomatic and radiographic improvement are also unclear. While culture conversion implies microbiological success, it does not reflect, nor has been correlated with, how the patient functions or feels, thus is not accepted by the United States Food and Drug Administration (FDA) as a primary end-point for clinical trials, further supported by guidance on developing drugs in NTM [7–10].

In a 1-day Patient-Centered Outcomes Research Institute (PCORI)-funded research priorities conference for NTM, which included patients, caregivers, patient advocates, clinical experts, and researchers, developing a composite measure of disease activity (CMDA), encompassing symptoms, health-related quality of life, functional measures, laboratory and radiographic results, was identified as a priority by participants [11]. Because outcome measures are not collected or evaluated consistently across all MAC-PD studies, this impedes our ability to compare results between studies, combine studies for meta-analyses, assess potential biases between different outcomes, and accumulate enough data to develop a valid, reliable and responsive CMDA [12–18]. Establishing the best available instruments for a minimum core outcome measurement instrument set, which can be consistently collected across future MAC-PD clinical trials, can facilitate the development of a CMDA for MAC-PD. In order to identify a core outcome measurement instrument set, or how to best measure outcomes (*i.e.* St George's Respiratory Questionnaire *versus* Quality of Life – bronchiectasis), it is necessary to identify and agree upon which outcome domains, or what types of outcomes (*i.e.* microbiology, radiology) are critical to include [19, 20].

We present an international consensus-based set of minimum core outcome domains (CODs) for MAC-PD clinical trials using a modified Delphi consensus methodology through collaboration with stakeholders including people living with MAC-PD, friends/family members, clinicians, researchers, and nonprofit, educational and federal research funding organisations. This is the first step in facilitating development of a CMDA for MAC-PD, which could promote patient-centred decisions regarding treatment of MAC-PD.

## Methods

We conducted a three-round international consensus process using a modified Delphi consensus methodology to develop COD for MAC-PD [21, 22]. Given the similarity of MAC-PD to other chronic inflammatory diseases, as opposed to acute infections where an intervention's efficacy and effectiveness are often more clearly defined (*i.e.* clearance of blood cultures, in-hospital mortality for bacteraemia), we modelled our approach after what has been done successfully in rheumatology and other specialties managing chronic inflammatory diseases to identify core outcome domains [23, 24].

### Delphi consensus process participant recruitment

**FIGURE 1:**
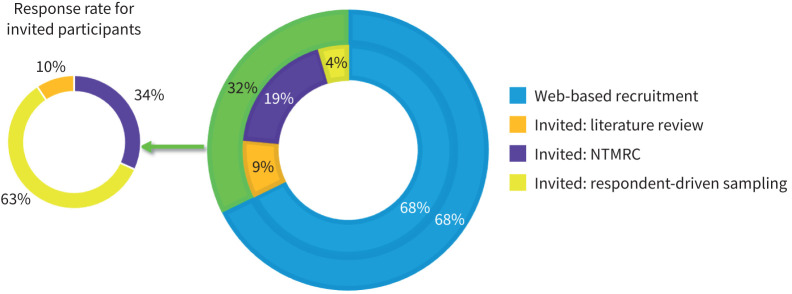
Participant recruitment sources and response rates for invited participants. NTMRC: Nontuberculous Mycobacteria Research Consortium.

To provide an international consensus, we utilised guidance provided by Outcome Measures in Rheumatology, PCORI and the Agency for Healthcare Research and Quality to identify stakeholder roles for inclusion as participants [23–26]. We identified the following stakeholder roles for inclusion: 1) MAC-PD researchers; 2) MAC-PD clinicians; 3) representatives from organisations that fund MAC-PD research; 4) people living with MAC-PD; and 5) family members or friends of people living with MAC-PD. For stakeholder roles 1–3, we included all first or senior authors on MAC-PD manuscripts or guidelines, NTM Research Consortium (NTMRC) members, in addition to NTM Network European Trials group (www.ntmnet.org/) and NTM Info & Research (NTMir) (https://ntminfo.org/) listservs [27]. For stakeholder roles 4–5, we used web-based recruitment in collaboration with NTMir listservs, patient support and education groups. In addition, international collaborators provided information for this study with survey links *via* listservs or websites to expand international enrolment for all stakeholder roles. We used respondent-driven sampling, where participants could recommend an additional individual while completing the round 1 survey (figure 1) [28]. We invited all identified stakeholders to participate *via* email, with a brief description of the Delphi consensus process and participation requirements, in addition to an information sheet and authorisation, where they could indicate their willingness to participate.

### Delphi consensus process methodology

All three survey rounds were administered electronically and were available in five languages (English, French, Japanese, Korean and Spanish). We developed and administered round 1 surveys through DelphiManager (COMET Initiative, Liverpool, UK; www.comet-initiative.org/delphimanager/) and round 2 and 3 surveys were developed and administered through REDCap electronic data capture tools hosted at Oregon Health & Science University (Portland, OR, USA) [29]. At the start of all survey rounds, we provided participants with the goals of the Delphi consensus process.

Initial outcome domains were selected based on prior work including the PCORI-funded research priorities conference for NTM, the FDA's Patient-Focused Drug Development Initiative meeting on NTM, which included patients, caregivers, patient advocates, clinical experts, and researchers, to identify preliminary outcome domains for MAC-PD research [10, 11]. In addition, we considered results of surveys conducted at the 2015 NTMRC meeting and by NTMir, a nonprofit organisation advocating for patients with pulmonary NTM, which evaluated patient preferences for treatment outcomes [30].

We provided participants with a description of outcome domains during each round (table 1). We asked participants to rate each outcome domain, without regard to the availability, feasibility or validity of a measurement instrument, using the Grading of Recommendations Assessment, Development and Evaluation scale: not important (1–3); important, but not critical (4–6); critical (7–9); unable to score (0) [31]. We defined inclusion in the COD as a consensus of the combined participants with ≥70% rating the domain as critical with a score of ≥7, and ≤15% of responders identifying the domain as not important (score ≤3), per guidelines [23, 25, 32]. If domains did not meet COD inclusion criteria in round 1 or 2, they were included in subsequent rounds. If domains met COD inclusion criteria, they were removed from subsequent rounds.

**TABLE 1:** Outcome domains and descriptions provided to participants during the international consensus process

Outcome domain	Description
**Microbiology^#^**	Sputum or bronchoalveolar lavage mycobacterial cultures, including antibiotic resistance
**Chest imaging^#^**	Radiographic or computed tomography scans of lungs
**Symptoms^#^**	*e.g.* cough, fatigue, fevers, chills, night sweats, shortness of breath, and other symptoms that make someone feel poorly
**Mental health**	Impact of MAC pulmonary disease on a person's psychological and emotional wellbeing (*e.g.* anxiety, depression)
**Biomarkers^#^**	A biological measure found in blood, body fluid or tissue that can be objectively measured to identify MAC pulmonary disease and its response to therapy
**Treatment burden^#^**	How the treatment for MAC pulmonary disease impacts people (time spent, number of pills, cost, changes in schedules, treatment adherence or ability to take all medications)
**Physical function^#^**	How MAC pulmonary disease impacts a person's ability to perform different functions, from caring for oneself (bathing, cooking meals, cleaning, climbing stairs) to activities a person enjoys (walking, exercising, playing sports, dancing, sewing, painting)
**Social function**	How MAC pulmonary disease impacts a person's ability to fulfil their role during social activities or in relationships with friends and family (*e.g.* avoidance of or discomfort going out in public due to cough)
**Role function**	How MAC pulmonary disease impacts a person's ability to fulfil their role at work, within their family or other relationships (quit a job or cut down on work hours, no longer able to perform childcare duties)
**Vitality/energy^#^**	How MAC pulmonary disease affects the amount of physical energy available to a person
**Treatment side-effects^#^**	Side-effects related to MAC pulmonary disease medications or pulmonary hygiene (*e.g.* rash, nausea, vomiting, diarrhoea, fatigue, increased cough)
**Disease recurrence^#^^,^^¶^**	Two or more positive respiratory cultures for MAC after finishing a treatment course for MAC
**Mortality^¶^**	Death during a MAC treatment course or a specified time after completing a MAC treatment course

MAC: *Mycobacterium avium* complex. ^#^: met inclusion criteria for core outcome domain set; ^¶^: newly recommended domains from round 1 added to subsequent rounds.

### Round 1

The round 1 survey included the following 11 outcome domains: symptoms, microbiology, treatment side-effects, chest imaging, physical function, treatment burden, vitality/energy, biomarkers, role function, mental health and social function (table 1). Participants were asked to use the above Delphi consensus process methodology to rate each outcome domain. We asked participants to recommend any additional outcome domains *via* a free-text field, and to provide comments regarding the domains. In addition, we collected demographic information including language preference, name, age, gender, race, ethnicity, stakeholder role, country, institution, preferred contact information, years of experience with MAC-PD in the selected stakeholder role, and potential conflicts of interest (supplementary material S1). People living with MAC-PD were asked additional questions including presence of cavitary disease, need for intravenous antibiotics to treat their MAC-PD, and MAC-PD disease recurrence.

We initiated the round 1 survey in English on 31 March 2023 with NTMRC members, followed by expansion to remaining participants on 8 June 2023, when language translations were available. We sent reminder emails and invitations to potential participants suggested through respondent-driven sampling each week. Round 1 closed on 20 September 2023. Participants were asked to contact the study team if they required clarifications, if additional support was needed to complete the survey, or if they wanted to withdraw from the study.

### Rounds 2 and 3

We invited all participants who completed round 1 to participate in round 2 and round 3. We provided histograms, stratified by stakeholder role, for prior round ratings for each outcome domain not meeting inclusion criteria in prior rounds. In addition, we provided each participant's individual prior ratings for outcome domains. We asked round 2 and 3 participants to re-rate the remaining outcome domains and rate any newly included outcome domains, following the same methodology as round 1. Rounds 2 and 3 were open between 31 May 2024 and 26 October 2024 and between 7 November 2024 and 28 February 2025, respectively. We sent participants who did not respond weekly reminders for up to 6 weeks, then every 2 weeks until the round closed.

### Statistical analysis

We used SAS version 9.4 (2013; Cary, NC, USA) to summarise participant demographics and outcome domain ratings. Stakeholder roles were combined into two groups: 1) people living with MAC-PD/family members or friends of people living with MAC-PD; 2) clinicians/researchers/representatives of funding organisations, to describe continent representation. Due to lack of probabilistic sampling, comparative statistics were not performed [26, 33].

## Results

### Study population

Round 1 included 306 participants representing 17 countries with a majority (72.5%) participating through web-based recruitment (table 2, figure 1). Most were women (73.5%) and White (81.0%). Over half of participants were people living with MAC-PD (57.2%), with 31.1% reporting clinician and 9.2% researcher as their primary role in working with MAC-PD. Years of MAC-PD experience within stakeholder roles varied with 36.9% reporting ≥10 years and 30.1% reporting 1–4 years. Most participants (n=266, 86.9%) reported no potential conflicts of interest (supplementary material S2).

**TABLE 2:** Characteristics of *Mycobacterium avium* Complex Core Outcomes Research Delphi consensus process participants

**Stakeholder role**	
Clinician who cares for people with MAC-PD	95 (31.1)
Researcher who studies MAC-PD	28 (9.2)
Representative from an organisation that funds MAC-PD research	≤5 (≤1.6)
Person with MAC-PD	175 (57.2)
Family member or friend of a person living with MAC-PD^#^	≤5 (≤1.6)
**Age group years**	
18–29	≤5 (≤1.6)
30–39	40 (13.1)
40–49	43 (14.1)
50–59	42 (13.7)
60–69	95 (31.0)
70–79	67 (21.9)
≥80	14 (4.6)
**Gender** ^¶^	
Woman	225 (73.5)
Man	80 (26.1)
**Race, ethnicity** ^+^	
American Indian or Alaska Native	≤5 (≤1.6)
Asian	41 (13.4)
Black or African American	≤5 (≤1.6)
Hispanic or Latinx	11 (3.6)
Native Hawaiian or Pacific Islander	≤5 (≤1.6)
White	248 (81.0)
Prefer not to respond	≤5 (≤1.6)
Unknown	≤5 (≤1.6)
**Experience with MAC-PD years**	
<1	33 (10.8)
1–4	92 (30.1)
5–9	62 (20.3)
≥10	113 (36.9)

Data are presented as n (%). Total respondents n=306. MAC-PD: *Mycobacterium avium* complex pulmonary disease. ^#^: 66.7% lived with their family member or friend living with MAC-PD; ^¶^: no participants identified as transgender or nonbinary; ^+^: participants were allowed to select as many race, ethnicity categories as they identified.

Most people living with MAC-PD were aged ≥60 years (83.4%) and women (94.1%), with 42.9% living with MAC-PD for ≥5 years. Most reported bronchiectasis (90.3%) or COPD (10.9%) as underlying lung conditions; however, 89.5% of those who reported COPD also reported bronchiectasis. Of people living with MAC-PD, 29.4% were told they had cavitary disease by a physician; 28.8% reported at least one MAC-PD recurrence after negative cultures; and 14.1% reported intravenous antibiotics to treat MAC-PD.

Four continents were represented by participants in the people living with MAC-PD/family members or friends of people living with MAC-PD combined stakeholder group and five continents were represented by participants in the clinicians/researchers/representatives of funding organisations combined stakeholder group in round 3 (table 3), per guidelines [23, 26].

**TABLE 3:** Region of *Mycobacterium avium* Complex Core Outcomes Research Delphi consensus process participants

	Round 1		Round 2^#^		Round 3^¶^	
	Person with MAC-PD/ family member or friend of a person with MAC-PD	Clinicians/researchers/representatives of funding organisations	Person with MAC-PD/ family member or friend of a person with MAC-PD	Clinicians/researchers/representatives of funding organisations	Person with MAC-PD/ family member or friend of a person with MAC-PD	Clinicians/researchers/representatives of funding organisations
**Participants**	306		197		173	
**Continent**						
Africa	0 (0.0)	≤5 (≤4.0)	0 (0.0)	0 (0.0)	0 (0.0)	0 (0.0)
Asia	≤5 (≤2.8)	29 (23.0)	≤5 (≤4.3)	18 (22.2)	≤5 (≤4.8)	13 (19.1)
Australia/Oceania	≤5 (≤2.8)	≤5 (≤4.0)	≤5 (≤4.3)	≤5 (≤6.2)	≤5 (≤4.8)	≤5 (≤7.4)
Europe	7 (3.9)	19 (15.1)	≤5 (≤4.3)	13 (16.1)	≤5 (≤4.8)	12 (17.6)
North America	165 (91.7)	74 (58.7)	108 (93.1)	48 (59.3)	97 (93.3)	43 (63.2)
South America	0 (0.0)	≤5 (≤4.0)	0 (0.0)	≤5 (≤6.2)	0 (0.0)	≤5 (≤7.4)
Not reported	≤5 (≤2.8)	0 (0.0)	≤5 (≤4.3)	0 (0)	≤5 (≤4.8)	0 (0.0)

Data are presented as n or n (%). MAC-PD: *Mycobacterium avium* complex pulmonary disease. ^#^: n=6 requested withdrawal from the study between round 1 and round 2; ^¶^: n=1 requested withdrawal from the study between round 2 and round 3.

### Delphi consensus process round 1

**FIGURE 2:**
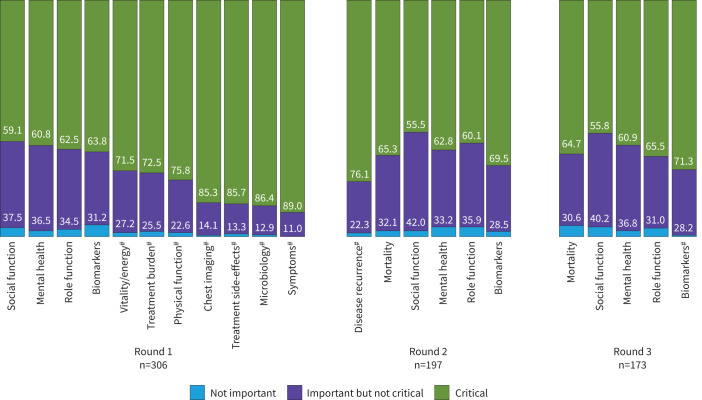
Distribution of outcome domain ratings by round. ^#^: domain met criteria for core outcome domain inclusion.

**FIGURE 3:**
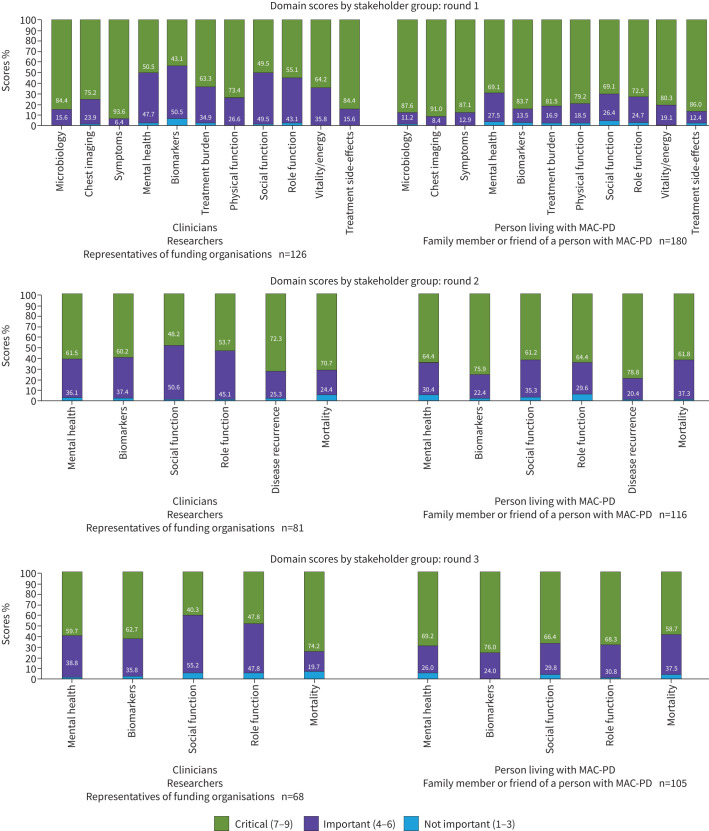
Distribution of domain scores by stakeholder group and round. MAC-PD: *Mycobacterium avium* complex pulmonary disease.

Symptoms, microbiology, treatment side-effects, chest imaging, physical function, treatment burden, and vitality/energy reached COD inclusion criteria during round 1, with ≥70% of participants rating these as critical, and they were excluded from subsequent rounds (figure 2, table 4). ≥70% people living with MAC-PD scored each outcome domain as critical. Apart from vitality/energy, none of the outcome domains reaching inclusion criteria had ≥5% of participants rating them as not important, and none of the outcome domains were rated as not important by ≥15% of participants*.* Overall, outcome domain ratings were similar between stakeholder groups, with greater diversity in median scores between stakeholder roles in biomarkers (median score 5–8; people living with MAC-PD/family members or friends of people living with MAC-PD providing higher ratings; 10.1% of clinicians rated biomarkers as not important); role function (median score 6–8; people living with MAC-PD providing higher ratings); social function (median score 5–7; people living with MAC-PD and researchers providing higher ratings); and treatment side-effects (median score 5–8; people living with MAC-PD and clinicians providing higher ratings) (table 4, figure 3). In addition, there was less variability in domain ratings in those with fewer years of experience with MAC-PC, with ≥70% of participants with <1 year of experience rating all domains as critical (supplementary material S4). Additional outcome domains of mortality and disease recurrence were recommended by participants for consideration in subsequent rounds.

**TABLE 4:** Distribution of round 1 outcome domain ratings by stakeholder group

	Score	Scored as not important (1–3)	Scored as critical (7–9)
**Microbiology**			
Clinicians	9 (4–9)	0.0	86.6
Researchers	9 (5–9)	0.0	77.8
People living with MAC-PD	9 (2–9)	1.3	88.0
Funding representatives	9 (9–9)	0.0	100.0
Family or friends	9 (6–9)	0.0	80.0
Total	9 (2–9)	0.7	86.5
**Chest imaging**			
Clinicians	8 (3–9)	1.2	74.7
Researchers	8 (5–9)	0.0	81.5
People living with MAC-PD	9 (2–9)	0.6	91.3
Funding representatives	9 (9–9)	0.0	100.0
Family or friends	9 (6–9)	0.0	80.0
Total	9 (2–9)	0.7	85.3
**Symptoms**			
Clinicians	8 (5–9)	0.0	92.8
Researchers	8 (5–9)	0.0	88.9
People living with MAC-PD	9 (4–9)	0.0	87.6
Funding representatives	9 (8–9)	0.0	100.0
Family or friends	8 (6–9)	0.0	80.0
Total	8 (4–9)	0.0	89.3
**Mental health**			
Clinicians	6 (2–9)	2.4	49.4
Researchers	7 (4–9)	0.0	51.9
People living with MAC-PD	7 (1–9)	3.7	71.0
Funding representatives	6 (5–8)	0.0	33.3
Family or friends	6 (5–9)	0.0	40.0
Total	7 (1–9)	2.9	61.8
**Biomarkers**			
Clinicians	6 (3–9)	10.1	38.0
Researchers	6 (4–9)	0.0	48.0
People living with MAC-PD	8 (2–9)	3.5	82.8
Funding representatives	5 (5–7)	0.0	33.3
Family or friends	8 (6–9)	0.0	75.0
Total	8 (2–9)	5.1	64.8
**Treatment burden**			
Clinicians	7 (3–9)	2.4	56.6
Researchers	7 (2–9)	3.6	71.4
People living with MAC-PD	8 (2–9)	1.9	82.7
Funding representatives	7 (7–9)	0.0	100.0
Family or friends	8 (7–9)	0.0	100.0
Total	7 (2–9)	2.1	74.4
**Physical function**			
Clinicians	7 (3–9)	1.2	72.3
Researchers	7 (5–9)	0.0	74.1
People living with MAC-PD	8 (2–9)	2.5	79.6
Funding representatives	7 (6–9)	0.0	66.7
Family or friends	8 (5–9)	0.0	60.0
Total	8 (2–9)	1.8	76.4
**Social function**			
Clinicians	6 (3–9)	2.4	44.6
Researchers	7 (4–9)	0.0	51.9
People living with MAC-PD	7 (2–9)	4.9	70.4
Funding representatives	5 (4–6)	0.0	0.0
Family or friends	7 (6–9)	0.0	60.0
Total	7 (2–9)	3.6	60.0
**Role function**			
Clinicians	6 (3–9)	4.8	49.4
Researchers	7 (4–9)	0.0	51.9
People living with MAC-PD	8 (2–9)	3.1	72.7
Funding representatives	7 (6–7)	0.0	66.7
Family or friends	6 (4–9)	0.0	25.0
Total	7 (2–9)	3.2	63.0
**Vitality/energy**			
Clinicians	7 (3–9)	3.6	59.0
Researchers	7 (4–9)	0.0	59.3
People living with MAC-PD	8 (3–9)	0.6	82.2
Funding representatives	7 (6–9)	0.0	66.7
Family or friends	7 (6–9)	0.0	80.0
Total	7 (3–9)	1.4	73.0
**Treatment side-effects**			
Clinicians	8 (4–9)	0.0	91.6
Researchers	7 (5–9)	0.0	70.4
People living with MAC-PD	8 (1–9)	1.2	88.3
Funding representatives	5 (5–7)	0.0	33.3
Family or friends	7 (3–9)	20.0	60.0
Total	8 (1–9)	1.1	86.4

Data are presented as median (range) or %. MAC-PD: *Mycobacterium avium* complex pulmonary disease.

### Delphi consensus process round 2

The round 2 survey included 197 participants (64.4% of those who responded to round 1); 103 did not respond, and six requested study withdrawal between round 1 and round 2. Of the stakeholder roles, 82.1% of researchers, 66.7% of representatives from funding organisations, 20.0% of friends or family members, 59.0% of clinicians and 66.7% of people living with MAC-PD completed round 2. The following outcome domains were included in round 2: biomarkers, role function, mental health, social function, and additionally recommended outcome domains mortality and disease recurrence. Disease recurrence was the only domain reaching COD inclusion criteria during round 2 with 76.1% of participants scoring it as critical*.* An increase in the proportion of participants rating biomarkers and mental health as critical was observed, whereas the proportion of participants rating social function and role function as critical decreased.

### Delphi consensus process round 3

The round 3 survey included 173 participants (56.5% of those who responded to round 1), with one additional participant withdrawing from the study between round 2 and round 3. All participants who completed round 3 also completed round 2. The following outcome domains were included: biomarkers, role function, mental health, social function and mortality. Biomarkers was the only outcome domain reaching COD inclusion criteria during round 3, with 71.3% of participants scoring it as critical*.* A small increase in the proportion of participants rating all domains except mortality as critical was observed; however, the changes in ratings between rounds 2 and 3 were minimal.

Following round 3, the final core outcome domains for MAC-PD selected by the Delphi consensus process were symptoms, microbiology, treatment side-effects, chest imaging, physical function, treatment burden, vitality/energy, disease recurrence, and biomarkers.

## Discussion

After a three-round multinational consensus process involving 306 stakeholders, participants identified nine CODs as being critical to future research on the management of MAC-PD. CODs represent the aspects of health or patient outcomes that are critical to assess when conducting research in a specific field or disease [23, 25, 26]. However, definitions of critical often differ by stakeholder role, encompassing healthcare structures and personal priorities, making it essential to include participants from various roles and locations [23, 24–26]. MAC-PD guidelines emphasise shared decision-making when approaching health decisions between people living with MAC-PD, their families and clinicians, yet there is sometimes a disconnect, often due to feasibility and trial logistics, between the clinical and research settings with regards to relevant outcomes [3, 4]. We included five stakeholder groups to ensure our CODs are relevant for researchers and funding organisations, in addition to people living with MAC-PD, their families and clinicians making individual treatment decisions.

A strength of our consensus process was the number of people living with MAC-PD (>50% of our sample) who participated. Despite lower response rates in round 2 and round 3, over half of our participants were people living with MAC-PD for every round and we maintained representation from three or more continents in all rounds. Prior consensus processes have not consistently incorporated patients and family members or friends, with patient representation noted in only 16–40% of systematic reviews [34, 35]. The involvement of people living with a health condition of interest has impacted COD selection in multiple other fields, highlighting the importance of their inclusion in the consensus process [36, 37].

Stakeholder groups rated most domains similarly, and ratings were fairly stable between rounds; however, differences were noted, probably reflecting diverse experiences by role. Biomarkers had the greatest difference in score by stakeholder group, with people living with MAC-PD and family members or friends of people living with MAC-PD providing higher ratings and clinicians and researchers rating these lower. Biomarkers would be an attractive option for patients and families, especially if they replaced sputum samples. Although sputum cultures are the current primary measure for diagnosis and treatment success, they are challenging to obtain, especially if productive cough resolves during treatment [3, 4, 11]. Because biomarkers often lack sensitivity and specificity, and a well-validated biomarker has not yet been established for MAC-PD, we suspect consideration of feasibility or scientific skepticism by clinicians and researchers may have contributed to lower ratings [38]. However, a greater focus on patient-centred outcomes, emphasised by FDA guidance, may also have resulted in differential ratings by stakeholder group [7–10]. Side-effects were also rated higher by clinicians and people living with MAC-PD, reflecting the challenges of long-term antibiotic treatment and high numbers of early treatment discontinuation [3, 4, 6, 39]. Notably, the social health domains of role function and social function were not included in the final COD [23]. Participant comments around these domains suggested they were linked to symptoms, such as cough and fatigue, which may have resulted in prioritisation of symptoms over other domains with conceptual overlap. In addition, mental health did not reach COD inclusion criteria, despite higher rates of depression and anxiety in this population [40]. Comments from clinicians, researchers and people living with MAC-PD regarding mental health acknowledged various contributing factors and the difficulty attributing any changes specifically to MAC-PD, and we suspect this is why it was not chosen as an independent domain. Furthermore, mortality did not meet criteria for COD inclusion, with 5% of participants rating it as not important. This may reflect that most people living with MAC-PD had been doing so for ≥5 years at the time of the round 1 survey, leading to prioritisation of other domains. Given the indolent nature of MAC-PD and long duration of therapy, the ideal timing to determine treatment impact on mortality is unknown, with 3–5 years of follow-up often required to observe mortality [16, 41]. Extended follow-up can be logistically challenging for both researchers and funding organisations to implement, suggesting that feasibility may have also been considered here.

Our study has limitations. Nearly half of participants did not respond to surveys through round 3, with the largest drop between rounds 1 and 2. We suspect that this was due to the extended time, driven by institutional delays and the need to change software platforms, between round 1 and 2. Although attrition differed by stakeholder roles, round 1 domain scores were similar between those who did and did not respond to round 2 and round 3 (supplementary material S3). A potential conflict of interest was reported by 13.1% of participants. Domain scores were also similar between those reporting conflicts and those who did not, except for biomarkers, where the median score of those with a conflict fell into the important but not critical category, and the median score of those without a conflict fell into the critical category (supplementary material S2). In addition, a diagnosis of MAC-PD was self-reported by participants and could result in misclassification. Though we met pre-specified criteria for continent representation, our numbers outside of North America were relatively low, especially in people living with MAC-PD, which may impact global generalisability [26, 35]. We also had no people living with MAC-PD and few clinicians and researchers from South America or Africa, which may be influenced by the higher rates of tuberculosis compared to NTM in these continents, in addition to competing priorities embedded in health system, social and policy structures, which can impact engagement in a consensus process [42–44]. The number of language translations we could provide was also restricted by budget constraints, limiting further global engagement. In addition, 94.1% of people living with MAC-PD were women. The higher proportion of women is not entirely unexpected, given disease epidemiology and published study participation; however, we must acknowledge the limited representation of men in this stakeholder role may bias domain scores [1, 2, 45].

### Conclusions

In an international consensus process, researchers, clinicians, representatives from funding organisations, family members, friends, and people living with MAC-PD identified nine minimum outcome domains (symptoms, microbiology, treatment side-effects, chest imaging, physical function, treatment burden, and vitality/energy, disease recurrence, biomarkers) for MAC-PD research. Identification and dissemination of the COD set will guide next steps in development of a core outcome measurement instrument set and research priorities for measurement instruments, in addition to facilitating the development of a CMDA for MAC-PD, further promoting patient-centred decisions regarding treatment of MAC-PD.

## Supplementary material

**Please note:** supplementary material is not edited by the Editorial Office, and is uploaded as it has been supplied by the author. Appendix 1 00636-2025.SUPPLEMENT Appendix 2 00636-2025.SUPPLEMENT2 Appendix 3 00636-2025.SUPPLEMENT3 Appendix 4 00636-2025.SUPPLEMENT4
