# Supplementary material for: Core outcome domains for Mycobacterium avium complex pulmonary disease: a MACCOR study
Source: ERJ Open Res. 2025 Dec 22;11(6):00636-2025. doi: 10.1183/23120541.00636-2025 (PMC12720154; doi:10.1183/23120541.00636-2025)
Supplement: Supplementary file 1 [file 00636-2025.SUPPLEMENT.pdf]

MACCOR

Mycobacterium avium complex Core Outcomes Research (MACCOR)

Core Outcome Domains Survey

Registration

Name ________________________

E-Mail address __________________________

Confirm Email __________________________

Please select the stakeholder role that best describes the majority of your time in relation to pulmonary MAC disease

- Clinician who cares for people with MAC pulmonary disease
- Researcher who studies MAC pulmonary disease
- Representative from an organization that funds MAC pulmonary disease research
- Person with MAC pulmonary disease
- Family member or friend of a person living with MAC pulmonary disease

[Stakeholder specific questions]

Clinician who cares for people with MAC pulmonary disease

How long have you been managing patients with MAC pulmonary disease?

- Less than 1 Year
- 1-4 Years
- 5-9 Years
- 10 Years or more
- I do not manage patients with MAC pulmonary disease

Institution or company: ____

Researcher who studies MAC pulmonary disease

What type of research do you do? Select all that apply

- Clinical
- Basic science
- Translational

How long have you been working in MAC pulmonary disease research?

- Less than 1 Year
- 1-4 Years
- 5-9 Years
- 10 Years or more
- I do not work in MAC pulmonary disease research

Institution or company: ____

Representative from an organization that funds MAC pulmonary disease research

How long have you been working in an organization that funds MAC pulmonary disease research?

- Less than 1 Year
- 1-4 Years
- 5-9 Years
- 10 Years or more
- I and not a representative from an organization that funds MAC pulmonary disease research

Institution or company: ____

Person with MAC pulmonary disease

How many years has it been since you were first diagnosed with MAC pulmonary disease?

- Less than 1 Year
- 1-4 Years
- 5-9 Years
- 10 Years or more
- I have not been diagnosed with a Mycobacterium avium complex (MAC) lung infection

Have you ever been told by your MAC provider that you have cavitary MAC pulmonary disease?

- Yes
- No

Have you had recurrence of your MAC pulmonary disease (completed 12 or more months of antibiotic therapy for MAC pulmonary disease with negative cultures followed by new sputum cultures positive for MAC)?

- Yes
    - i. How many times have you had MAC pulmonary disease recurrence? ____________________
- No

Have you ever required IV or intravenous antibiotics to treat your MAC pulmonary disease?

- Yes
- No

What type of lung problems have you been told by a doctor that you have? Select all that apply

- Cystic Fibrosis
- Bronchiectasis
- Chronic obstructive pulmonary disease (COPD)/emphysema
- Pulmonary fibrosis
- Other _______________________________________

Family member or friend of a person living with MAC pulmonary disease

Do you live with a family member or friend who has MAC pulmonary disease?

- Yes
- No
- I do not have a friend or family member with MAC pulmonary disease

Age

- 18-29 years
- 30-39 years
- 40-49 years
- 50-59 years
- 60-69 years
- 70-79 years
- ≥ 80 years

Race, Ethnicity – select all that apply

- American Indian or Alaska Native
- Asian
- Black or African American
- Hispanic or Latino
- Native Hawaiian or Other Pacific Islander
- White
- Other ________________________
- Prefer not to respond

Gender

- Woman
- Man
- Transgender man
- Transgender woman
- Non-binary
- Other ______________________
- Prefer not to respond

Country

____

Please list any potential conflicts of interest, including financial, employment or personal. If none, please type none.

We would like to ensure a wide range of opinions are reflected in this consensus process. If there is an individual you feel should be invited to participate, please list their email address and select their stakeholder role:

____

- Clinician
- Researcher
- Representative from an organization that funds MAC pulmonary disease research
- Person with MAC pulmonary disease
- Family member or friend of a person living with MAC pulmonary disease

Please select the top 5 most important symptoms of MAC pulmonary disease:

- Fatigue
- Cough
- Shortness of breath
- Chest congestion
- Mucus/sputum production
- Hemoptysis or coughing up blood
- Passing out or loss of consciousness from coughing
- Throat clearing
- Fevers
- Chills
- Night sweats
- Sleep disruption/problems sleeping
- Weight loss
- Poor appetite
- Taste changes
- Smell changes
- Memory problems
- Mental fogging
- Depressed mood
- Symptoms of anxiety
- Trouble carrying and lifting items
- Trouble completing tasks for work or as part of a family
- Decreased participation in social activities

Consensus process goals: Currently, there is not a standardized approach for evaluating how MAC pulmonary disease affects an individual. Physicians and researchers are using different outcomes or measures in studies, which makes it difficult to compare study results. We aim to identify the core outcome domains that are essential for MAC pulmonary disease research and care. These outcomes are called **core outcome domains**. Please rate the importance of each domain (1 least important, 9 most important) to assess how well-being changes with treatment for MAC pulmonary disease. Please rate domains without regard to the availability, feasibility, or validity of a measurement instrument. A future study will evaluate how each outcome domain is or will be measured.

Your survey responses will not be linked to your name.

Microbiology: sputum or bronchoalveolar lavage mycobacterial cultures

1	2	3	4	5	6	7	8	9	0
Not Important			Important but not Critical				Critical		Unable to Score

Chest imaging: X-rays or computed tomography (CT or CAT) scans of lungs

1	2	3	4	5	6	7	8	9	0
Not Important			Important but not Critical				Critical		Unable to Score

Symptoms: for example, cough, fatigue, fevers, chills, night sweats, shortness of breath, and other symptoms that make someone feel poorly.

1	2	3	4	5	6	7	8	9	0
Not Important			Important but not Critical				Critical		Unable to Score

Mental health: impact of MAC pulmonary disease on a person's psychological and emotional well-being (for example, anxiety, depression).

1	2	3	4	5	6	7	8	9	0
Not Important			Important but not Critical				Critical		Unable to Score

Biomarkers: a biological measure found in blood, body fluid or tissue that can be objectively measured to identify MAC pulmonary disease and its response to therapy.

1	2	3	4	5	6	7	8	9	0
Not Important			Important but not Critical				Critical		Unable to Score

Treatment burden: how the treatment for MAC pulmonary disease impacts people (time spent, number of pills, cost, changes in schedules).

1	2	3	4	5	6	7	8	9	0
Not Important			Important but not Critical				Critical		Unable to Score

Physical function: how MAC pulmonary disease impacts one's ability to perform different functions [for example, from caring for oneself (bathing, cooking meals, cleaning, climbing stairs) to activities one enjoys (walking, exercising, playing sports, dancing, sewing, painting)].

1	2	3	4	5	6	7	8	9	0
Not Important			Important but not Critical				Critical		Unable to Score

Social function: how MAC pulmonary disease impacts one's ability to fulfill one's role during social activities or in relationships with friends and family (for example, avoidance of or discomfort going out in public due to cough).

1	2	3	4	5	6	7	8	9	0
Not Important			Important but not Critical				Critical		Unable to Score

Role function: how MAC pulmonary disease impacts one's ability to fulfill one's role at work, within one's family or other relationships (quit a job or cut down one work hours, no longer able to perform childcare duties).

1	2	3	4	5	6	7	8	9	0
Not Important			Important but not Critical				Critical		Unable to Score

Vitality/Energy: how pulmonary MAC effects the amount of physical energy available to a person.

1	2	3	4	5	6	7	8	9	0
Not Important			Important but not Critical				Critical		Unable to Score

Treatment Side effects: Side effects related to MAC pulmonary disease medications or pulmonary hygiene (for example, rash, nausea, vomiting, diarrhea, fatigue, increased cough).

1	2	3	4	5	6	7	8	9	0
Not Important			Important but not Critical				Critical		Unable to Score

Please consider other domains that may be important to assess in MAC pulmonary disease. If there is a domain not listed previously that we should consider, please click "Add Outcome Row" then write in and rate the outcome
below:____

We would like to list members who participated in the Delphi panel in future publications, however this is optional, and you can participate and remain anonymous. If you would like to be listed as a participant, please provide your name and degree, if applicable. Your survey responses will not be linked to your name.

____

Round 2 Introduction

Thank you for completing the surveys for the Mycobacterium avium complex Core Outcomes Research (MACCOR) study. The Round 2 survey is now available and should take less than 5 minutes to complete.

Based on your responses, the following outcome domains will be included as core outcome domains:

- Microbiology: sputum or bronchoalveolar lavage mycobacterial cultures, including antibiotic resistance.
- Chest imaging: X-rays or computed tomography (CT or CAT) scans of lungs
- Symptoms: for example, cough, fatigue, fevers, chills, night sweats, shortness of breath, and other symptoms that make someone feel poorly.
- Treatment burden: how the treatment for MAC pulmonary disease impacts people (time spent, number of pills, cost, changes in schedules, treatment adherence or ability to take all medications).
- Physical function: how MAC pulmonary disease impacts one's ability to perform different functions [for example, from caring for oneself (bathing, cooking meals, cleaning, climbing stairs) to activities one enjoys (walking, exercising, playing sports, dancing, sewing, painting)].
- Vitality/Energy: how pulmonary MAC effects the amount of physical energy available to a person.
- Treatment Side effects: Side effects related to MAC pulmonary disease medications or pulmonary hygiene (for example, rash, nausea, vomiting, diarrhea, fatigue, increased cough).

Round 2 includes newly recommended domains and domains that enough participants did not rate as Critical.

You will be able to see your prior rating and the prior rating from everyone who participated in Round 1.

Questions Round 2

Consensus process goals: Currently, there is not a standardized approach for evaluating how MAC pulmonary disease affects an individual. Physicians and researchers are using different outcomes or measures in studies, which makes it difficult to compare study results. We aim to identify the core outcome domains that are essential for MAC pulmonary disease research and care. These outcomes are called core outcome domains. Please rate the importance of each domain (1 least important, 9 most important) to assess how well-being changes with treatment for MAC pulmonary disease. Please rate domains without regard to the availability, feasibility, or validity of a measurement instrument. A future study will evaluate how each outcome domain is or will be measured.

Mental health: impact of MAC pulmonary disease on a person's psychological and emotional well-being (for example, anxiety, depression).

1	2	3	4	5	6	7	8	9	0
Not Important			Important but not Critical				Critical		Unable to Score

Biomarkers: a biological measure found in blood, body fluid or tissue that can be objectively measured to identify MAC pulmonary disease and its response to therapy.

1	2	3	4	5	6	7	8	9	0
Not Important			Important but not Critical				Critical		Unable to Score

Social function: how MAC pulmonary disease impacts one's ability to fulfill one's role during social activities or in relationships with friends and family (for example, avoidance of or discomfort going out in public due to cough).

1	2	3	4	5	6	7	8	9	0
Not Important			Important but not Critical				Critical		Unable to Score

Role function: how MAC pulmonary disease impacts one's ability to fulfill one's role at work, within one's family or other relationships (quit a job or cut down one work hours, no longer able to perform childcare duties).

1	2	3	4	5	6	7	8	9	0
Not Important			Important but not Critical				Critical		Unable to Score

Mortality: death during a MAC treatment course or a specified time after completing a MAC treatment course.

1	2	3	4	5	6	7	8	9	0

Not Important	Important but not Critical	Critical	Unable to Score

Disease Recurrence: Two or more positive respiratory cultures for MAC after finishing a MAC treatment course.

1	2	3	4	5	6	7	8	9	0
Not Important			Important but not Critical				Critical		Unable to Score

Comments

Please provide any comments you think would be important for us know.

Thank you

Thank you for completing the surveys for the Mycobacterium avium complex Core Outcomes Research (MACCOR) study. We value the time it takes to answer these questions. Your answers will help future patients, as we are better able to describe the impacts of MAC and directions for future research.

If you have any questions or comments about your experience, please email MACCOR@ohsu.edu.

Round 2 Introduction

Thank you for completing the surveys for the Mycobacterium avium complex Core Outcomes Research (MACCOR) study. The Round 2 survey is now available and should take less than 5 minutes to complete.

Based on your responses, the following outcome domains will be included as core outcome domains:

- Microbiology: sputum or bronchoalveolar lavage mycobacterial cultures, including antibiotic resistance.
- Chest imaging: X-rays or computed tomography (CT or CAT) scans of lungs
- Symptoms: for example, cough, fatigue, fevers, chills, night sweats, shortness of breath, and other symptoms that make someone feel poorly.
- Treatment burden: how the treatment for MAC pulmonary disease impacts people (time spent, number of pills, cost, changes in schedules, treatment adherence or ability to take all medications).
- Physical function: how MAC pulmonary disease impacts one's ability to perform different functions [for example, from caring for oneself (bathing, cooking meals, cleaning, climbing stairs) to activities one enjoys (walking, exercising, playing sports, dancing, sewing, painting)].
- Vitality/Energy: how pulmonary MAC effects the amount of physical energy available to a person.
- Treatment Side effects: Side effects related to MAC pulmonary disease medications or pulmonary hygiene (for example, rash, nausea, vomiting, diarrhea, fatigue, increased cough).

Round 2 includes newly recommended domains and domains that enough participants did not rate as Critical.

You will be able to see your prior rating and the prior rating from everyone who participated in Round 1.

Questions Round 3

Consensus process goals: Currently, there is not a standardized approach for evaluating how MAC pulmonary disease affects an individual. Physicians and researchers are using different outcomes or measures in studies, which makes it difficult to compare study results. We aim to identify the core outcome domains that are essential for MAC pulmonary disease research and care. These outcomes are called core outcome domains. Please rate the importance of each domain (1 least important, 9 most important) to assess how well-being changes with treatment for MAC pulmonary disease. Please rate domains without regard to the availability, feasibility, or validity of a measurement instrument. A future study will evaluate how each outcome domain is or will be measured.

Mental health: impact of MAC pulmonary disease on a person's psychological and emotional well-being (for example, anxiety, depression).

1	2	3	4	5	6	7	8	9	0
Not Important			Important but not Critical				Critical		Unable to Score

Biomarkers: a biological measure found in blood, body fluid or tissue that can be objectively measured to identify MAC pulmonary disease and its response to therapy.

1	2	3	4	5	6	7	8	9	0
Not Important			Important but not Critical				Critical		Unable to Score

Social function: how MAC pulmonary disease impacts one's ability to fulfill one's role during social activities or in relationships with friends and family (for example, avoidance of or discomfort going out in public due to cough).

1	2	3	4	5	6	7	8	9	0
Not Important			Important but not Critical				Critical		Unable to Score

Role function: how MAC pulmonary disease impacts one's ability to fulfill one's role at work, within one's family or other relationships (quit a job or cut down one work hours, no longer able to perform childcare duties).

1	2	3	4	5	6	7	8	9	0
Not Important			Important but not Critical				Critical		Unable to Score

Mortality: death during a MAC treatment course or a specified time after completing a MAC treatment course.

1	2	3	4	5	6	7	8	9	0

Not Important	Important but not Critical	Critical	Unable to Score

Comments

Please provide any comments you think would be important for us know.

Thank you

Thank you for completing the surveys for the Mycobacterium avium complex Core Outcomes Research (MACCOR) study. We value the time it takes to answer these questions. Your answers will help future patients, as we are better able to describe the impacts of MAC and directions for future research.

If you have any questions or comments about your experience, please email MACCOR@ohsu.edu.
