# Supplementary material for: Core outcome domains for Mycobacterium avium complex pulmonary disease: a MACCOR study
Source: ERJ Open Res. 2025 Dec 22;11(6):00636-2025. doi: 10.1183/23120541.00636-2025 (PMC12720154; doi:10.1183/23120541.00636-2025)
Supplement: Supplementary file 2 [file 00636-2025.SUPPLEMENT2.pdf]

Appendix 2: Conflicts of Interest

Most participants, (N=266, 86.9%) reported no potential conflicts of interest. Of those who reported a potential conflict of interest, 4 reported employment, 9 reported serving on advisory boards, 6 reported serving as consultants, 6 reported being investigators for pharmaceutical sponsored clinical trials, 3 reported receiving funding for lectures or speakers bureaus, and 13 reported a conflict with an undisclosed role. Companies included AN2, Crestone, Genentech, Insmed, MannKind, Oricula Therapeutics, Spero and various non-profit organizations.

Appendix 2 Table: Median Scores of Round 1 Outcome Domain Ratings by Participants who Reported a Conflict of Interest and Participants who Reported No Conflicts

	Conflict of Interest Reported n=40	No Conflict of Interest Reported n=266
	**Median Score (range)**	**Median Score (range)**
Microbiology	9 (6-9)	9 (2-9)
Chest Imaging	7 (3-9)	9 (2-9)
Symptoms	9 (6-9)	9 (6-9)
Mental Health	7 (4-9)	7 (1-9)
Biomarkers	5 (2-9)	7 (2-9)
Treatment Burden	7 (6-9)	8 (2-9)
Physical Function	8 (5-9)	8 (2-9)
Social Function	8 (4-9)	7 (2-9)
Role Function	7 (4-9)	7 (2-9)
Vitality/Energy	8 (5-9)	8 (3-9)
Treatment Side Effects	8 (4-9)	8 (1-9)
