# Supplementary material for: Core outcome domains for Mycobacterium avium complex pulmonary disease: a MACCOR study
Source: ERJ Open Res. 2025 Dec 22;11(6):00636-2025. doi: 10.1183/23120541.00636-2025 (PMC12720154; doi:10.1183/23120541.00636-2025)
Supplement: Supplementary file 3 [file 00636-2025.SUPPLEMENT3.pdf]

Appendix 3: Median Scores of Round 1 Outcome Domain Ratings by Round 3 Completion vs. Round 2 Completion vs. No Response/Withdrawal Between Round 1 and 2.

	Completed Round 3 n=173	Completed Round 2 n=197	No Response/Withdrawal Between Round 1 and 2 n=109
	**Median Score (range)**	**Median Score (range)**	**Median Score (range)**
Microbiology	9 (2-9)	9 (2-9)	9 (4-9)
Chest Imaging	9 (2-9)	9 (2-9)	9 (4-9)
Symptoms	8 (5-9)	8 (4-9)	9 (4-9)
Mental Health	7 (1-9)	7 (1-9)	7 (1-9)
Biomarkers	7 (1-9)	7 (2-9)	7 (2-9)
Treatment Burden	8 (2-9)	8 (2-9)	8 (3-9)
Physical Function	8 (3-9)	8 (2-9)	8 (2-9)
Social Function	7 (2-9)	7 (2-9)	7 (2-9)
Role Function	7 (2-9)	7 (2-9)	8 (2-9)
Vitality/Energy	7 (3-9)	7 (3-9)	8 (4-9)
Treatment Side Effects	8 (4-9)	8 (4-9)	8 (1-9)
