# Supplementary figures and images for: Core outcome domains for Mycobacterium avium complex pulmonary disease: a MACCOR study
Source: ERJ Open Res. 2025 Dec 22;11(6):00636-2025. doi: 10.1183/23120541.00636-2025 (PMC12720154; doi:10.1183/23120541.00636-2025)

**Appendix 4:** Distribution of Domain Scores by Years of Experience with MAC-PD

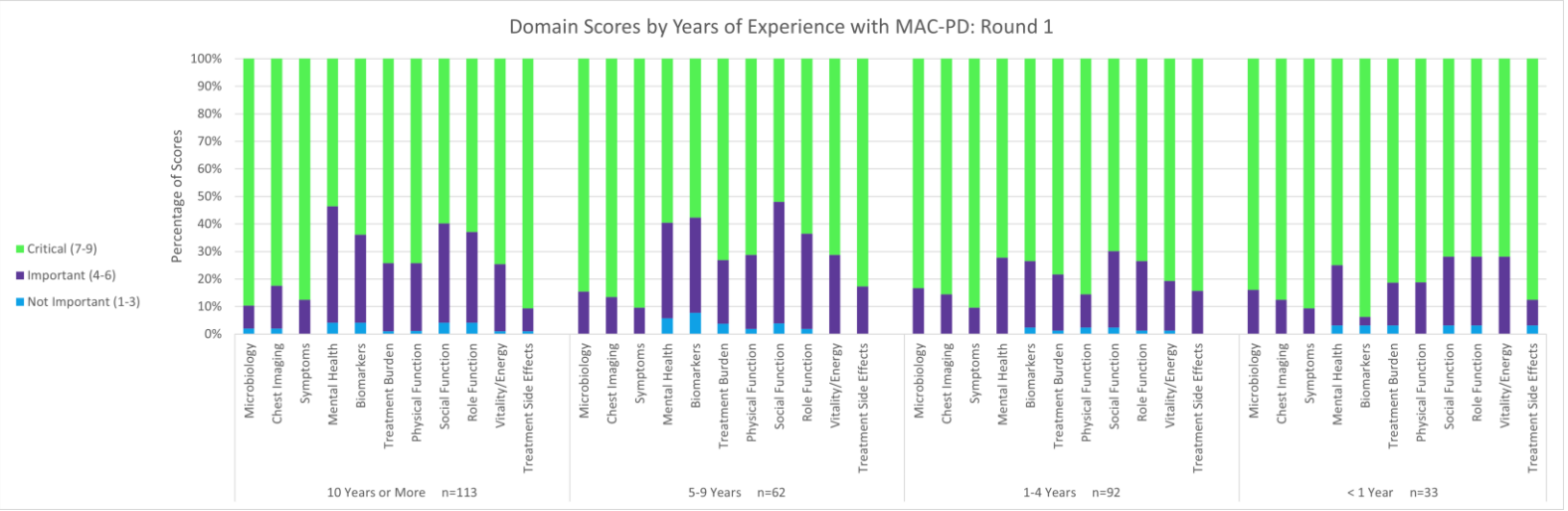

Supplement: Supplementary file 4 [file 00636-2025.SUPPLEMENT4.pdf]
